# Supplementary material for: The reverse quantum limit and its implications for unconventional quantum oscillations in YbB12
Source: Nat Commun. 2024 Feb 21;15:1607. doi: 10.1038/s41467-024-45801-2 (PMC10881586; doi:10.1038/s41467-024-45801-2)
Supplement: Supplementary file 1 — Supplementary Information [file 41467_2024_45801_MOESM1_ESM.pdf]

Supplementary Information

Supplementary Note 1: Methods

Growth and Structural Characterization

YbB_{12} single crystals were grown via the traveling solvent method under Ar gas atmosphere in a laser diode floating zone furnace (Crystal Systems Corp.) at the Platform for the Accelerated Realization, Analysis and Discovery of Interface Materials (PARADIM) user facility at Johns Hopkins University using a similar procedure as previously documented [1] and described in the Methods section. A representative diffraction pattern in Supplementary Fig. 1 provides evidence of the crystal quality. The crystallographic lattice parameters, relevant data collection information, and the corresponding refinement statistics are provided in Supplementary Table 1.

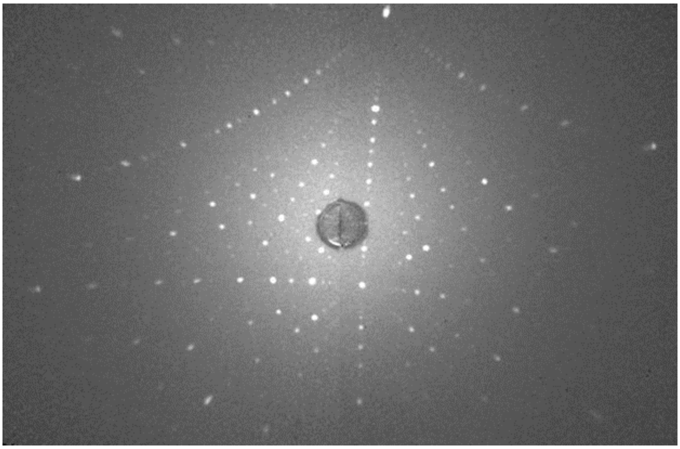

Supplementary Figure 1 A single crystal domain on edge oriented close to the [011] crystallographic axis.

Supplementary Table 1 Crystallographic and refinement parameters for YbB_{12}.

a (Å)	7.4752(2)
V (Å^3)	417.70(3)
Z	4
T (K)	297(2)
θ-range (deg)	3.7 - 68.1
μ (mm^{-1})	11.93
Measured reflections	14740
Independent reflections	273
R_{int}	0.072
$\Delta\rho_{\max}$ (e/Å^3)	1.23
$\Delta\rho_{\min}$ (e/Å^3)	1.51
Extinction coefficient	0.0088(5)
Data / restraints / parameters	273 / 0 / 7
R_1 ($F^2 > 2\sigma(F^2)$)	0.010 [a]
wR_2 (F^2)	0.020 [b]

[a] $R_1 = \frac{\sum \left| |F_o| - |F_c| \right|}{\sum |F_o|}$

[b] $wR_2 = \left(\frac{\sum w(F_o^2 - F_c^2)^2}{\sum w(F_o^2)^2} \right)^{1/2}$

Supplementary Note 2: Physical Property Characterization

The zero-field resistivity of the YbB_{12} sample used for the high field studies reported in the main text was first characterized using a low-frequency AC resistance bridge and a standard four-wire configuration in a Quantum Design Physical Property Measurement System (PPMS). The resistivity increased by over 4 orders of magnitude when cooled from 300K to 0.5K, as shown in Supplementary Fig. 2. The insulating behavior stems from the opening of small gaps (E_a = 2.7 meV and E_a = 4.7 meV, inset). These gaps are thought to arise from the hybridization between conduction electrons and largely localized f-electrons [2]. Below ∼3K the resistivity exhibits a "plateau" seen in other YbB_{12} samples, often attributed to a metallic surface [1, 3, 4].

Supplementary Fig. 3 shows the magnetic susceptibility as a function of temperature from 1.8K to 350K measured with magnetic fields of 0.1T and 5T. The data were obtained on a Quantum Design Magnetic Properties Measurement System (MPMS3) equipped with a 7T magnet. Above ∼170K the susceptibility exhibits Curie-Weiss behavior (Supplementary Fig. 3b) giving a Curie temperature of -134.8±1.3K (-123.6±2.3K) and effective moments per Yb of 4.6±0.3μ_B (4.4±0.3μ_B) for an applied field of 0.1T (5T). These values are consistent with a nominal +3 valence for Yb (effective moment of 4.54 μ_B) [1]. The susceptibility exhibits a maximum at ∼75K, minimum at ∼17K, and a low temperature increase which is suppressed by field. The maximum is characteristic of Kondo singlet formation and the temperature of the susceptibility maximum is proportional to the Kondo temperature [5]. The low temperature increase in susceptibility is indicative of magnetic impurities [1].

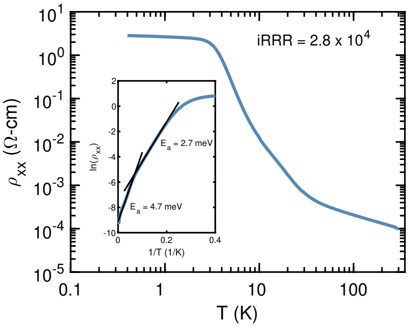

Supplementary Figure 2 Resistivity as a function of temperature in zero magnetic field. The resistivity increases by a factor of $2.8{\times}10^4$ from 300K to 0.5K owing to the opening of gaps (inset).

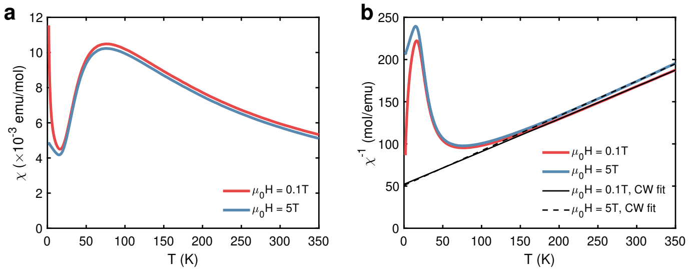

Supplementary Figure 3 (a) Magnetic susceptibility as a function of temperature measured with fields of 0.1T (red) and 5T (blue). (b) Inverse susceptibility with Curie-Weiss fits.

Specific heat measurements (Supplementary Fig. 4) were collected in a PPMS calorimeter using a quasiadiabatic thermal relaxation technique with a ^{3}He insert before and after the sample was cut and polished for the pulsed field experiments. Before polishing the mass of the crystal was $\sim$5 mg and after polishing the mass was $\sim$1.1 mg. Both measurements show a low temperature upturn, often attributed to a Schottky contribution [4]. In both cases a linear extrapolation of C/T yields a Sommerfeld coefficient of 1.3$\pm$0.2 mJ/molK^2. This value is smaller than values reported in the literature [4], but of the same order of magnitude.

Supplementary Note 3: Basis of the Reverse Quantum Limit Scenario in YbB_{12}

It is instructive to consider how the Kondo insulator ground state arises by considering a simplified form of the Anderson lattice model in one dimension [6]. We consider hybridization between a conduction electron band of the form $\varepsilon_k = t\cos ak$ and a narrow f-electron band $\varepsilon_f = 0$ for which a hybridization potential V gives rise to reconstructed conduction and valence bands of

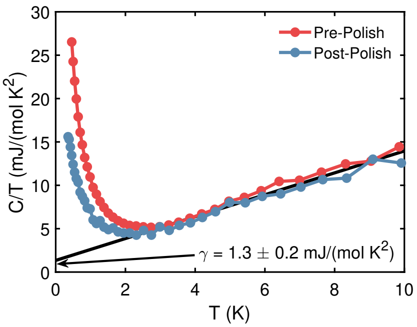

Supplementary Figure 4 Heat capacity as a function of temperature before and after polishing. A linear extrapolation of C/T gives an intercept of $\gamma = 1.3 \pm 0.2$ mJ/molK^2.

the form

$$E_{C,V} - \mu = \frac{t}{2}\cos ak \pm \sqrt{\frac{t^2}{4}\cos^2 ak + V^2}, \tag{1}$$

where μ is the chemical potential and a is the lattice periodicity. For simplicity, we have neglected the electron spin. This dispersion gives rise to an indirect gap Δ with the conduction band minimum located at the point $ak = \pi$, and the valence band maximum located at the point $ak = 0$ (see schematic in Supplementary Fig. 5). We perform a Taylor expansion of Equation (1) about these points, considering the limit $V < \mid \frac{t^2}{4}\cos^2 ak \mid$, and obtain

$$\begin{aligned} E_{C,V} - \mu &\approx \pm\frac{V^2}{t}\frac{1}{\cos ak} \\ &\approx \pm\frac{V^2}{t}\left(1 + \frac{a^2k^2}{2} + \ldots\right). \end{aligned} \tag{2}$$

Equation (2) can be rewritten in the form

$$E_{C,V} - \mu \approx \pm\left(\Delta + \frac{\hbar^2k^2}{2m^*}\right), \tag{3}$$

to lowest order in k, where $\Delta = V^2/t$ and $m^* = t\hbar^2/V^2a^2$. Note that since $V < t$, Δ is expected to much smaller than t while m^* values are expected to be larger than those of the unhybridized conduction electron band. Experimentally, the effective masses we observe are approximately twenty times larger than those of the unhybridized conduction bands in LuB_{12} [7], which has completely filled f-electron shells.

The formation of hybridized conduction and valence bands can be generalized to higher dimensions. If ε_k is isotropic in two or three dimensions with respect to the top or bottom of ε_k, then the hybridization gives rise to a Mexican hat dispersion form, which has been postulated to represent the dispersion in the vicinity of the X point in SmB_6 [8, 9]. For a more general anisotropic forms of ε_k, E_C will exhibit minima and E_V will exhibit maxima at

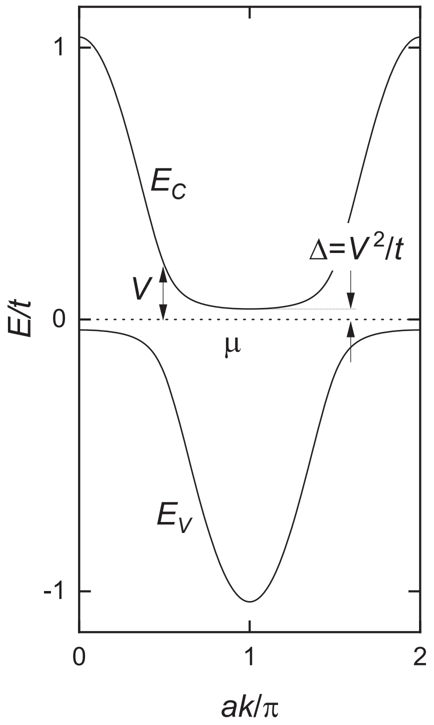

Supplementary Figure 5 Schematic of the hybridization in a periodic lattice.

distinctly different symmetry-related points in the Brillouin zone. These would become, respectively, electron and hole pockets were the chemical potential to be increased or decreased. Such a scenario has indeed been suggested in YbB_{12} on the basis of electronic structure calculations [7].

Since the dispersion is parabolic to lowest order in the vicinity of each of the conduction band minima and valence band maxima, it is useful to consider the orbital area A_k orthogonal to an applied magnetic field instead of k, so that

$$E_{C,V} - \mu \approx \pm\left(\Delta + \frac{\hbar^2 A_k}{2\pi m^*}\right), \tag{4}$$

where m^* now refers to the orbital effective mass. Equation (2) in the main text is obtained by considering E_C, and then applying the Bohr-Sommerfeld quantization rule: $A_k = \frac{2\pi eB}{\hbar}(\nu + \frac{1}{2})$ for fermions and including the Zeeman energy. For the valence band, we have

$$E_V^{\uparrow\downarrow} - \mu = -\Delta - \frac{\hbar e}{m^*}B\left(\nu + \frac{1}{2}\right) \mp \frac{1}{2}g^*\mu_B B. \tag{5}$$

Since the Kondo gap in YbB_{12} is expected to be indirect, the minima in the conduction band and the maxima in the valence band are located at different points in k-space [7]. Under such circumstances, there is no requirement for m^* to be the same for E_C and E_V, although (for simplicity) we have assumed this to be the case in constructing the schematic for Fig. 4a of the main text.

Supplementary Note 4: Reverse Quantum Limit Criterion

Beginning from Eq. (2) in the main text, we have

$$E_C^{\uparrow} - \mu = \Delta - \frac{1}{2}g^*\mu_B B + \frac{\hbar e}{m^*}B\left(\nu + \frac{1}{2}\right). \tag{6}$$

Landau level crossings occur when $E_C^{\uparrow} = \mu$. Therefore,

$$\Delta = \left(\frac{1}{2}g^*\mu_B - \frac{\hbar e}{m^*}\left(\nu + \frac{1}{2}\right)\right)B. \tag{7}$$

Since the zero-field gap (Δ)and field are positive quantities, the above equality requires

$$\frac{1}{2}g^*\mu_B > \frac{\hbar e}{m^*}\left(\nu + \frac{1}{2}\right). \tag{8}$$

Focusing on the first Landau level ($\nu = 0$) and substituting $\mu_B = \frac{e\hbar}{2m_e}$ yields the criterion for the reverse quantum limit:

$$g^*\frac{m^*}{m_e} > 2. \tag{9}$$

Landau level diagrams for the reverse quantum limit and the other four high-field scenarios described in Fig. 4 of the main text are provided in Supplementary Fig. 6.

Supplementary Note 5: Landau Level Pinning to Insulator-Metal Transition in Reverse Quantum Limit

Landau level crossings occur when $E_C^{\uparrow} - \mu = 0$. If we assume g^* and m^* are independent of field we can write the following expression for Landau level crossings

$$0 = \Delta(B) - \frac{1}{2}g^*\mu_B B + \frac{\hbar e}{m^*}B(\nu + \frac{1}{2}) \tag{10}$$

We can simplify this expression by relating g^* to the insulator-metal transition field. To do this we consider that at Landau level crossings, the activation energy is zero, so

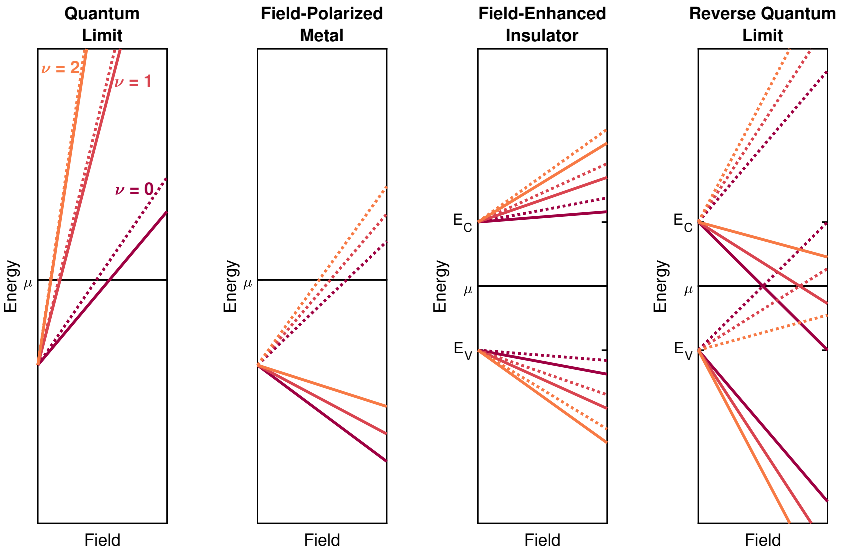

Supplementary Figure 6 Four high-field scenarios described in Fig. 4 of the main text corresponding to whether the material is gapped at the chemical potential and the relative size of the Zeeman and cyclotron energies. Colors correspond to the different Landau indices defined in the first panel and dashed/solid lines denote different spins.

$$2E_a = (E_C^{\uparrow} - \mu) - (E_V^{\downarrow} - \mu) = 0. \tag{11}$$

or equivalently,

$$2\Delta(B) - g^*\mu_B B_\nu + \frac{2\hbar e}{m^*} B_\nu(\nu + \frac{1}{2}) = 0 \tag{12}$$

Assuming $\Delta(B) = \Delta$ is a constant in the insulating state prior to the insulator-metal transition and that the insulator-metal transition occurs at a Landau level crossing corresponding to ν_{IM}, then we have

$$2\Delta - g^*\mu_B B_{IM} + \frac{2\hbar e}{m^*} B_{IM}(\nu_{IM} + \frac{1}{2}) = 0. \tag{13}$$

This expression relates the zero-field gap to the insulator-metal transition field and effective g factor. Subtracting Supplementary Eq. (12) and Supplementary Eq. (13) and reducing gives

$$(\nu - \nu_{IM})\frac{\hbar e}{m^*} = \frac{\Delta}{B_{IM}} - \frac{\Delta(B)}{B_\nu}. \tag{14}$$

When $\Delta(B)$ does not depend on field (i.e, $\Delta(B) = \Delta$ which applies in the insulating state) we have

$$(\nu - \nu_{IM})\frac{\hbar e}{m^*\Delta} = \frac{1}{B_{IM}} - \frac{1}{B_\nu} \tag{15}$$

where we now explicitly add the subscript ν to B to denote the field at which Landau level ν crosses the chemical potential. Since the left hand side of the equation is only weakly dependent on angle, in the reverse quantum limit Landau level indices are expected to be pinned to the insulator-metal transition as is shown in Fig. 3 of the main text.

We can also confirm this behavior by looking at the angular dependence of the quantum oscillation frequency in the insulating state because Supplementary Eq.(15) implies a constant quantum oscillation frequency which is independent of angle. Specifically, the quantum oscillation frequency assuming a constant gap is

$$|F| = \frac{m^*\Delta}{\hbar e}. \tag{16}$$

As shown in Section 3, $\Delta \propto 1/m^*$ so this expression predicts a quantum oscillation independent of mass and is an analogue to the familiar Onsager relation found in conventional metals. Within the resolution of our experiments, the quantum oscillation frequency in the insulating state determined from Landau level indexing (Supplementary Fig. 7) or Fourier transforms (Supplementary Fig. 8) is independent of angle.

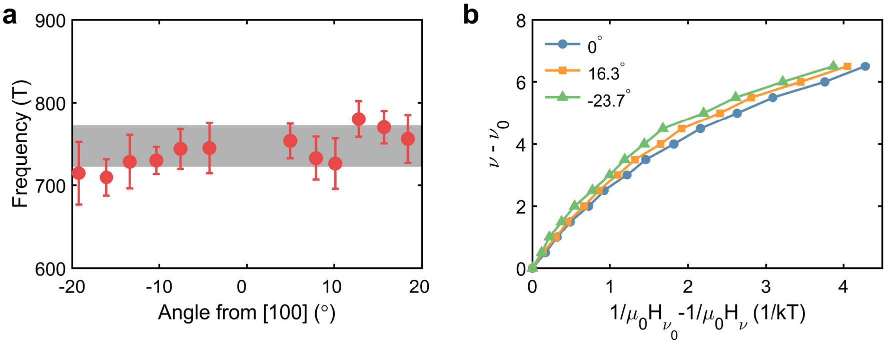

Supplementary Figure 7 (a) Quantum oscillation frequency determined from Landau level indexing in the insulating state as a function of angle has minimal variations over a wide angular range. (b) Landau index as a function of inverse field in the metallic state (both quantities are referenced to the first observed quantum oscillation according to Supplementary Eq.(14)). The angular dependence is related to anisotropies in the magnetization and Fermi surface.

When the hybridization gap changes with field (relevant in the metallic state), Supplementary Eq.(14) applies. In this situation we expect the Landau levels to be pinned to the insulator-metal transition close to the transition, with deviations at higher field owing to Δ having a field dependence. This explains the slight deviations from the angular dependence of the insulator-metal transition in the metallic state shown in Fig. 3 of the main text. These deviations are more clearly shown by plotting the Landau index as a function of inverse field at fixed angles (Supplementary Fig. 7). As we show in subsequent sections, this behavior is consistent with the field-dependent quantum

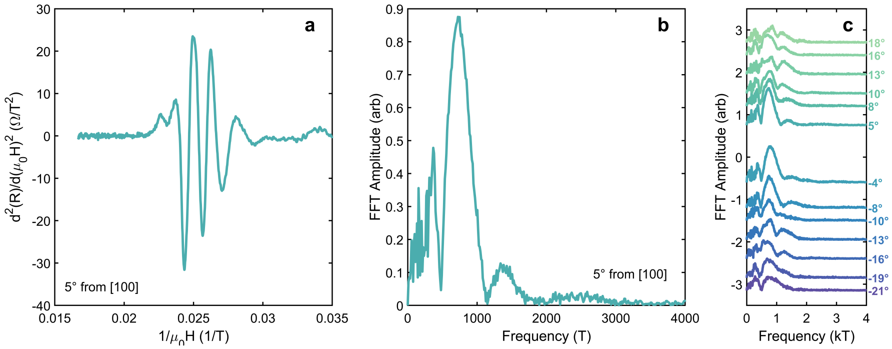

Supplementary Figure 8 (a) Second derivative of magnetoresistance with respect to inverse field with the field applied 5° from the [100] crystallographic axis in the [100]-[011] plane. (b) Fourier transform of the data shown in (a) indicating a dominant frequency of 740T with a harmonic. The Fourier transform amplitudes below 500T are likely artifacts. (c) Fourier transforms of the magnetoresistance reveal minimal variation in the dominant frequency with angle, in agreement with the angular dependence of the quantum oscillation frequency deduced from Landau level indexing. All data acquired at ∼650mK.

oscillation frequency in the metallic state and is related to anisotropies in the magnetization and Fermi surface.

Our SdH data in the insulating state of YbB_{12} indicates minimal variations in the quantum oscillation frequency with angle over a 40° range. This conclusion appears to be independent of whether we compute the quantum oscillation frequency directly from Landau level indices or with a Fourier transform (Fig. 5 of the main manuscript, Supplementary Figs. 7 and 8). The only other angular dependent measurements of SdH frequencies in YbB_{12} that we are aware of are described in Ref. [3]. In that work, a pronounced angular dependence in the SdH data with a frequency of ∼800 T for H applied parallel to [001] is reported. However, more recent work from the same group in Ref. [10] indicates a significantly smaller SdH frequency of ∼678 T for a magnetic field applied in the same direction. This newer reported frequency conflicts with the original reported value, but now agrees with the reported dHvA frequencies in both their works [3, 10] as well our SdH frequency. While we cannot provide a detailed explanation for the origin of this difference, we hypothesize it has to do with sample quality. The original report of the angular dependence of the SdH quantum oscillations in the insulating state of YbB_{12} had few, small amplitude oscillations [3] compared to later reports [11] and our own measurements. The number, and presence, of SdH oscillations seems to be intimately linked to sample quality. With so few oscillations, it can be difficult determine a frequency with high accuracy which could lead to the observed differences. This is clearly an important hypothesis to address with future angular dependent experiments.

Supplementary Note 6: Field-Dependent Frequency in Reverse Quantum Limit

Beginning from a generalization of Supplementary Eq. (6) where we do not assume a particular field dependence for the gap

$$E_C^{\uparrow} - \mu = \Delta(B) - \frac{1}{2}g^*\mu_B B + \frac{\hbar e}{m^*}B\left(\nu + \frac{1}{2}\right), \tag{17}$$

we impose the condition for quantum oscillations and solve for the Landau index

$$\nu + \frac{1}{2} = \frac{g^*\mu_B m^*}{2\hbar e} - \frac{\Delta(B)m^*}{\hbar e B}. \tag{18}$$

Given that the quantum oscillation frequency is related to the Landau index [12] via

$$F = \frac{d\nu}{d\frac{1}{B}} = -B^2\frac{d\nu}{dB}, \tag{19}$$

we find that in the reverse quantum limit

$$F = \frac{m^*}{\hbar e}\left(B\frac{d\Delta(B)}{dB} - \Delta(B)\right). \tag{20}$$

More generally, starting from

$$E_C^{\uparrow} - \mu = \Delta(B) - \frac{1}{2}g^*\mu_B B + \frac{\hbar e}{m^*}B\left(\nu + \frac{1}{2}\right), \tag{21}$$

but we let g^*, m^*, and Δ all vary with B. Then, we once again impose the condition for quantum oscillations and solve for the Landau index yielding

$$\nu + \frac{1}{2} = \frac{m^*(B)g^*(B)\mu_B}{2\hbar e} - \frac{\Delta(B)m^*(B)}{\hbar e B}. \tag{22}$$

Using the relation between the quantum oscillation frequency and Landau indices given by Supplementary Eq. (19), we find

$$F = \frac{1}{\hbar e}\Bigg(-m^*(B)\Delta(B) + B\left[m^*(B)\frac{d\Delta(B)}{dB} + \Delta(B)\frac{dm^*(B)}{dB}\right] - \frac{\mu_B}{2}B^2\left[m^*(B)\frac{dg^*(B)}{dB} + g^*(B)\frac{dm^*(B)}{dB}\right]\Bigg) \tag{23}$$

Since the B^2 term is small for the field ranges in these experiments, we can neglect the last term in this expression giving

$$F = \frac{1}{\hbar e}\left(-m^*(B)\Delta(B) + B\left[m^*(B)\frac{d\Delta(B)}{dB} + \Delta(B)\frac{dm^*(B)}{dB}\right]\right). \tag{24}$$

Then, because m^* experimentally scales linearly with field in the metallic state, a non-linear field dependence in the quantum oscillation frequency requires Δ to vary with field.

Supplementary Note 7: Relating Fermi Surface Area, Quantum Oscillation Frequency, and Landau Indices to Magnetization

As described in the main text, interpreting our experiments in terms of the reverse quantum limit model suggests the field-dependent quantum oscillation frequency in the metallic state occurs because the gap evolves as a non-linear function of field in the metallic state. A possible origin for a non-linear field dependence of the gap is that the $c-f$ hybridization responsible for the gap is altered by the magnetic field [5]. This possibility is consistent with partial f-electron polarization leading to non-linear magnetization in the field-induced metallic state [11, 13, 14]. The non-linear magnetization used throughout our analysis is shown in Supplementary Fig. 9.

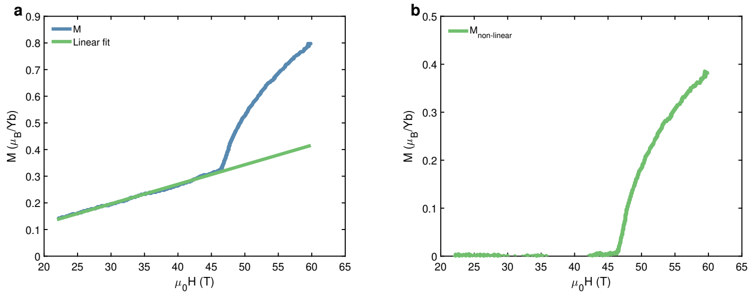

Supplementary Figure 9 (a) Magnetization of YbB_{12} with $\mathbf{H} \parallel [100]$ at T = 0.64K (blue). A linear fit to the magnetization in the insulating state (green) was used to obtain the non-linear magnetization. (b) The non-linear magnetization obtained by subtracting the linear fit from the total magnetization. Data taken from Ref. [11].

If this interpretation is correct, one expects the Fermi surface area measured by the quantum oscillations to be proportional to the non-linear portion of the magnetization corresponding to the partial f-electron polarization [5]. More specifically, since magnetization is the magnetic dipole moment per unit volume one expects the Fermi surface area to be proportional to the non-linear magnetization to the $\frac{2}{3}$ power for ellipsoidal pockets leading to

$$A = a\left(\frac{M(B)}{\mu_B}\right)^{2/3} \tag{25}$$

where A is the Fermi surface area, μ_B is the Bohr magneton, $M(B)$ is the field-dependent, non-linear magnetization, and a is a dimensionless constant

related to the degeneracy factor (see next section). Similar analyses have been performed in heavy fermion systems including UPt_3 [15].

Now we relate the non-linear magnetization to the Landau indices and quantum oscillation frequency. Landau level indices (ν) are related to the quantum oscillation frequency (F) according to

$$F = \frac{d\nu}{d\,(1/B)} \tag{26}$$

or, equivalently,

$$\nu = \frac{F}{B} + \nu_0 \tag{27}$$

where B is the magnetic field and ν_0 is a constant. We can then relate the Landau level indices to the extremal orbit area of the Fermi surface, A, using the Onsager relation [12],

$$F = \frac{\hbar}{2\pi e} A, \tag{28}$$

which yields

$$\nu = \frac{\hbar}{2\pi e}\frac{A}{B} + \nu_0. \tag{29}$$

Next, using the result from Supplementary Eq. (25), it is possible to directly relate the Landau indices to the non-linear component of the magnetization. This relationship is

$$\nu = \frac{\hbar}{2\pi e}\frac{1}{B} a \left(\frac{M(B)}{\mu_B}\right)^{2/3} + \nu_0. \tag{30}$$

Furthermore, using Supplementary Eq. (26) one can express the measured quantum oscillation frequency in terms of the non-linear magnetization.

$$F_{measured} = -B^2 \frac{d}{dB}\left[\frac{\hbar}{2\pi e}\frac{1}{B} a \left(\frac{M(B)}{\mu_B}\right)^{2/3}\right]. \tag{31}$$

The value of a given in the main text is fit by comparing our experimental quantum oscillation frequency in the high-field metallic state to the right-hand side of Supplementary Eq. (31) computed using the magnetization data from Ref. [11]. The Landau level indices are then extracted from Supplementary Eq. (30) using the same value of a and finding the value of ν_0 which gives the best agreement with the experimental magnetization given in Ref. [11]. Note, Supplementary Eq. (31) accounts for the effects of field-dependent quantum oscillations [16, 17].

Now, we relate the gap to the non-linear magnetization. From Supplementary Eq.(14)

$$\Delta(B) = \Delta \frac{B}{B_{IM}} - \nu \frac{\hbar e}{m^*} B. \tag{32}$$

Combining this condition with the relationship between the Landau indices and non-linear magnetization yields an expression for the gap after the onset of non-linear magnetization, i.e. the insulator-metal transition:

$$\Delta(B) = \Delta \frac{B}{B_{IM}} - \frac{\hbar^2}{2\pi m^*} a \left(\frac{M(B)}{\mu_B} \right)^{2/3}. \tag{33}$$

Supplementary Note 8: Estimating Carrier Density and Pocket Degeneracy

Since the Fermi surface area is known from Supplementary Eq. (25) it is possible to estimate the carrier concentration. Without knowing the exact shape of the Fermi surface, we first make a crude estimate for the carrier concentration assuming a spherical Fermi surface [12, 18]. In this case, the carrier concentration n is related to the Fermi surface area through

$$n = \frac{A^{3/2}}{3\pi^2}. \tag{34}$$

From Fig. 6 of the main text, the Fermi surface area near 60T is between $5 - 10\text{nm}^{-1}$, corresponding to a carrier concentration of $n \sim 10^{20} - 10^{21}\text{cm}^{-3}$ or $\sim 0.2 - 0.5$ carriers per unit cell. This value is consistent with carrier concentrations of other Kondo metals [19] and high field measurements we have performed on other YbB_{12} samples at similar temperatures (Supplementary Fig. 10).

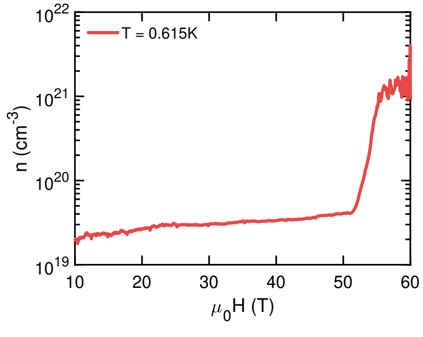

Supplementary Figure 10 High field carrier concentration at T = 0.615K obtained on a different sample of YbB_{12}.

Lastly, we express the constant a introduced in Supplementary Eq. (25) to the pocket degeneracy factor D, and in doing so provide an estimate for the pocket degeneracy. Our approach is to relate the magnetization contribution of the partially spin-polarized hybridized bands to the magnetization implied by the Landau level index in Supplementary Eq. (30).

Similar to Pauli paramagnetism [18], the magnetization per unit volume stemming from spin-polarization is given by

$$M = \frac{1}{2} g^* \mu_B D n \tag{35}$$

where D is the pocket degeneracy and n is the concentration of spin-polarized carriers. Accounting for spin degeneracy, n is related to the wavevector k through

$$n = \frac{k^3}{3\pi^2} \tag{36}$$

and k is related to the Landau index via

$$k^2 = \frac{2e}{\hbar} B \left(\nu + \frac{1}{2} \right). \tag{37}$$

Therefore, the magnetization is related to the Landau index through

$$M = \frac{1}{6\pi^2} g^* \mu_B \left(\frac{2e}{\hbar} B \left(\nu + \frac{1}{2} \right) \right)^{3/2}. \tag{38}$$

Substituting Supplementary Eq.(38) into Supplementary Eq. (30) and rearranging yields

$$D = \frac{6\pi^{7/2}}{g^* a^{3/2}}. \tag{39}$$

Using the value of the fit parameter $a \approx 3$ obtained from relating the measured frequency to the magnetization (i.e., Supplementary Eq. (31)) and $g^* \approx 2$, we find $D \approx 32$. This indicates YbB_{12} has a Fermi surface with high degeneracy, similar to what has been suggested in Ref. [7, 20]. It is also important to note that even without exact knowledge of the pocket shape, there can be a number of additional factors affecting this degeneracy factor estimate such as the saturated moment per Yb differing from μ_B [14], a change in the band Van Vleck contribution [2], and/or a field-dependence of g^*.

Supplementary Note 9: Determination of the Insulator-Metal Transition and Landau Level Indexing

Values for the insulator-metal transition field as a function of angle were taken from the contactless resistivity measurements because the onset of the metallic state corresponds to a large change in the slope of the TDO frequency (*e.g.*, Fig. 2 of main text). A precise determination of the insulator-metal transition was more difficult with the conventional resistivity measurements because of the dramatic change in the sample's resisitivity as a function of magnetic field: a current that would provide a sufficient signal-to-noise ratio to resolve the

IM transition would cause large Joule heating in the insulating state. The use of the insulator-metal transition field from the contactless resistance measurements, and the slight differences in sample alignment between the contactless resistance and conventional resisitivity measurements, is likely responsible for the small difference in the concavity of the the angular dependences of the insulator-metal transition and insulating state quantum oscillations shown in Fig. 3 of the main text.

The angular dependence of the quantum oscillations was determined by assigning Landau level indices and following the angular dependence of each Landau level. We found this to be more robust than Fourier analysis owing to the few oscillations in the insulating state. Indexing in this manner makes it apparent that there is some evolution of fine structure in the insulating and metallic oscillations as a function of angle. In cases where peak splitting was apparent, we would take the average position of the split peaks as the field assigned to that Landau index. The origin of the peak splitting is unclear. While it has been suggested to be a consequence of spin-splitting [11], there will also be contributions from the shape of the Fermi surface, g-factor anisotropy, and anisotropic f-electron polarization [14], which complicate the situation. The exact origin of the peak splitting does not impact the central claims of this work.

Supplementary Note 10: Lifshitz-Kosevich Analysis

According to Lifshitz-Kosevich theory [12], quantum oscillations possess a temperature dependent amplitude damping factor (R_T) of

$$R_T = \frac{\alpha \frac{T}{B} \frac{m^*}{m_e}}{\sinh\left(\alpha \frac{T}{B} \frac{m^*}{m_e}\right)}, \tag{40}$$

where

$$\alpha = \frac{2\pi^2 k_B m_e}{e\hbar}. \tag{41}$$

In order to isolate the amplitude of the quantum oscillations to determine m^* from Supplementary Eq.(40), it is necessary to account for the background signal. A simple polynomial subtraction proved to be sufficient for the TDO data, however the significant magnetoresistance in the insulating state complicates the analysis. Following Pippard [12, 21], we perform a background normalization [10] instead of a subtraction for the quantum oscillations in the insulating state, effectively assuming

$$\frac{\rho_{oscillatory}}{\rho_{background}} \sim R_T \frac{g_{oscillatory}(\mu)}{g_{background}(\mu)} \tag{42}$$

where ρ is resistivity, $g(\mu)$ is the density of states at the chemical potential. This normalization more properly accounts for temperature dependent scattering rates.

The results of this analysis are shown in Supplementary Fig. 11, along with data from LK analyses available in the literature [10, 11]. The effective masses of both the insulating and metallic states are in good agreement with those previously reported [3, 10, 11]. We also performed a measurement of the mass as a function of field in the metallic state at 18.6° from the [100] crystallographic axis in the [100]-[011] plane (Supplementary Fig. 12). When combined with the measurements along the [100] axis, these measurements reveal minimal anisotropy in the effective mass of the field-induced metallic state.

While we did not observe strong deviations from LK behavior, this could be because we could not reach sufficiently low temperatures. Moreover, the substantial background magnetoresistance in the insulating state makes subtle deviations from LK difficult to discern. It is worth noting that the data presented in Ref. [10] does show deviations from LK behavior in the SdH data. Although an alternative explanation is provided, it is possible the deviations could be caused by an excitonic insulator phase.

Supplementary Note 11: Gap Extraction with Parallel Conduction Model

To corroborate the gap closure results from the Arrhenius fits presented in Fig. 1 of the main text, we also analyzed the gap closure using a parallel conduction model that has previously been used to analyze transport in YbB_{12} [4]. The simplest model assumes the total conductance consists of two conduction channels, a constant channel often attributed to a metallic surface [4], and an

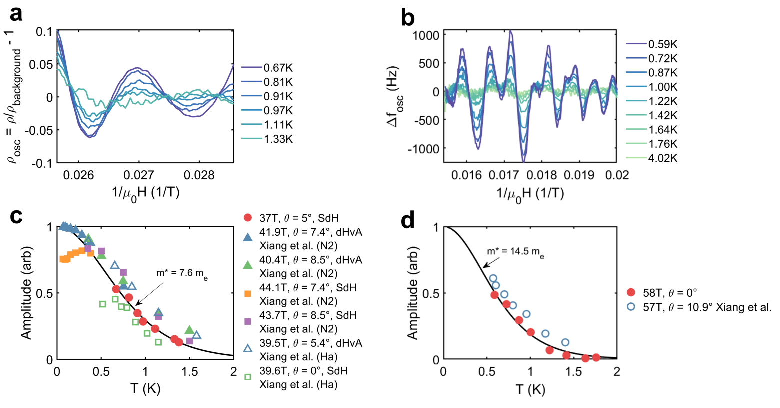

Supplementary Figure 11 Effective mass analysis near [100] in the (a,c) insulating and (b,d) metallic states based on Lifshitz-Kosevich theory. The temperature dependent amplitudes of the (a) insulating state oscillations were obtained by background normalization and the temperature dependent amplitudes of the (d) metallic state oscillations were obtained by background subtraction. The temperature dependent amplitudes of the (c) insulating and (d) metallic quantum oscillation are well described the Lifshitz-Kosevich formula. Comparisons are made to LK analyses from Ref. [10, 11]

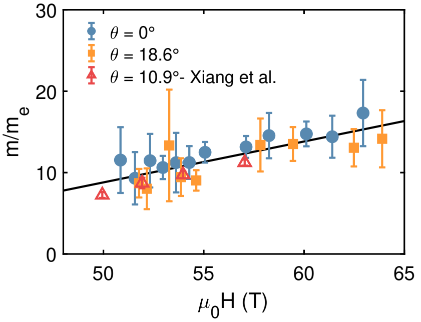

Supplementary Figure 12 Effective mass as a function of field at two different angles in the field-induced metallic state. At both angles the effective mass increases linearly with applied field. The effective mass exhibits minimal anisotropy. A comparison is made to data from Ref. [11]

activated channel. As such, we fit the conductance as a function of temperature at fixed fields to the model

$$G = A + G_0 \exp\left(-\frac{E_a}{T}\right) \tag{43}$$

where G is the total conductance, A is the constant conduction channel, G_0 is the activated channel's prefactor, and E_a is the activation energy. Supplementary Fig. 13 shows examples of fits to this model at fixed fields of 20T and 43T (applied parallel to [100]). Note, only the $E_a = 30K$ gap was included in this analysis because the larger gap (*e.g.*, Supplementary Fig. 2) has a negligible contribution to the total conductance in this temperature and field range.

In Supplementary Fig. 13a, two fits are shown to the experimental data. The blue fit treats E_a as a fit parameter. The red fit constrains E_a to a fixed value obtained by assuming the gap evolves linearly in field from its measured zero field value (Supplementary Fig. 2) through Zeeman splitting, i.e.

$$E_a(B) = \Delta - \frac{1}{2} g^* \mu_B B. \tag{44}$$

While there is minimal difference between these two fits at 20T (Supplementary Fig. 13a), the assumption of linear-in-field gap closure is unable to describe the data at high fields (Supplementary Fig. 13b). Even with the addition of a third conduction channel which has been used to describe a possible nodal semimetallic state in SmB_6 [22], such that

$$G = A + G_0 \exp\left(-\frac{E_a}{T}\right) + \beta T, \tag{45}$$

it is clear the high field data cannot be fit by models which assume the gap closes linearly with field. Moreover, the gap values extracted from treating the gap as a free parameter are substantially (4-5×) larger than those required were Supplementary Eq. (44) to apply over the entire field range.

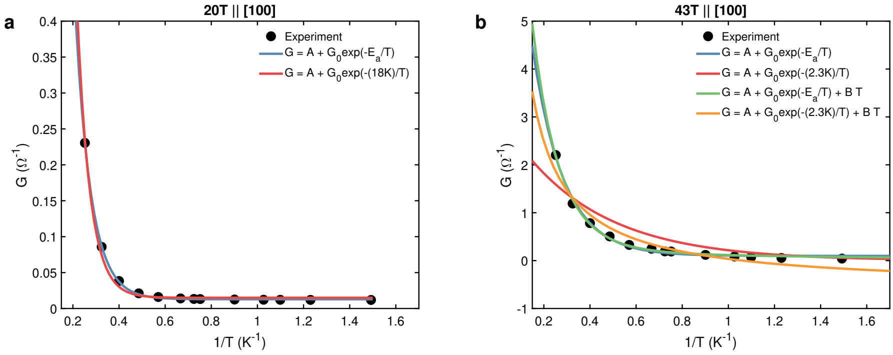

Supplementary Figure 13 Parallel conduction model fits to the conductance at fixed fields of (a) 20T and (b) 43T. In the lower field regime, the data is equally well fit with the gap considered to be a free parameter or constrained to take on a value assuming linear-in-field gap closure. The high field data cannot be described by linear-in-field gap closure.

Note, a comparison of the fits in Supplementary Fig. 13b with and without the third conduction channel indicate that both models capture the experimental data. Since inclusion of the linear term does not appreciably change the fitted gap value, it is excluded from subsequent analysis.

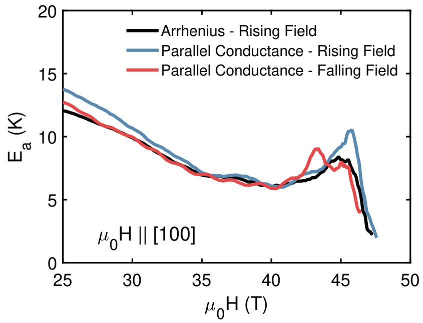

Supplementary Figure 14 A comparison of the high field behavior of the gap extracted from an Arrhenius analysis (rising field only) and parallel conduction fits (rising and falling fields). All methods give similar gap behavior and demonstrate the gap closes non-monotonically at high fields.

Having demonstrated that the gap closes non-linearly at high fields, we now confirm the results of our Arrhenius fit by comparing those findings to the high field dependence of the gap acquired from parallel conduction fits with the model describeed by Supplementary Eq. (45). As shown in Supplementary Fig. 14, the high field dependences of the gap acquired from the Arrhenius analysis and from the parallel conduction plots are in good agreement. This provides additional evidence that the gap does not monotonically close at high fields.

Supplementary Note 12: Gap Closure Along [011] Crystallographic Axis

In addition to examining the field-induced gap closure for fields applied along the [100] crystallographic axis, similar measurements were performed with field applied along the [011] crystallographic axis. Supplementary Fig. 15a shows the field-dependence of the magnetoresistance acquired at fixed temperature. This data possesses a similar field dependence to the [100] data shown in Fig. 1a of the main text. Quantum oscillations in the insulating state of YbB_{12} are also present in this measurement configuration at high fields, but their small amplitudes preclude a quantitative analysis. An Arrhenius analysis (Supplementary Fig. 15b) indicates gap closure with the field applied along [011] is similar to gap closure with the field along [100]. Supplementary Fig. 15c demonstrates this similarity by comparing the field-dependence of the gap when the field is applied parallel to [100] and [011] with fields normalized by the insulator-metal transition field. These data show that the gap closes non-linearly for fields applied along multiple crystallographic axes, consistent with the possibility of an excitonic insulator transition prior to the insulator-metal transition. Note, an Arrhenius analysis at lower field values for the [011] case was unreliable owing to an insufficient amount of high temperature data.

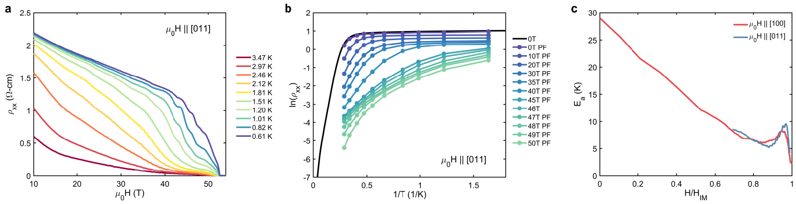

Supplementary Figure 15 (a) YbB_{12} magnetoresistance with the magnetic field applied along the [011] crystallographic axis. Along this direction the insulator-metal transition occurs at ∼54 T. (b) An Arrhenius analysis of the resistivity was used to obtain the evolution of the gap as a function of field in pulsed fields (PF). (c) The gap closure for fields along the [100] and [011] show similar deviations from linear gap closure when the field is normalized by the insulator-metal transition in each direction.

Supplementary Note 13: Checks for Field-Induced Heating Effects

Field-induced heating in pulsed field experiments can arise from induced Eddy currents or magnetocaloric effects [23]. Eddy current heating can be a substantial problem for metallic samples in pulsed fields, but can also be an issue in insulating samples owing to their metallic contacts.

Eddy current heating typically scales with H^2, so we performed a series of pulses with different maximum fields to check for field-induced heating effects. As shown in Supplementary Fig. 16, the field dependence of the resistivity

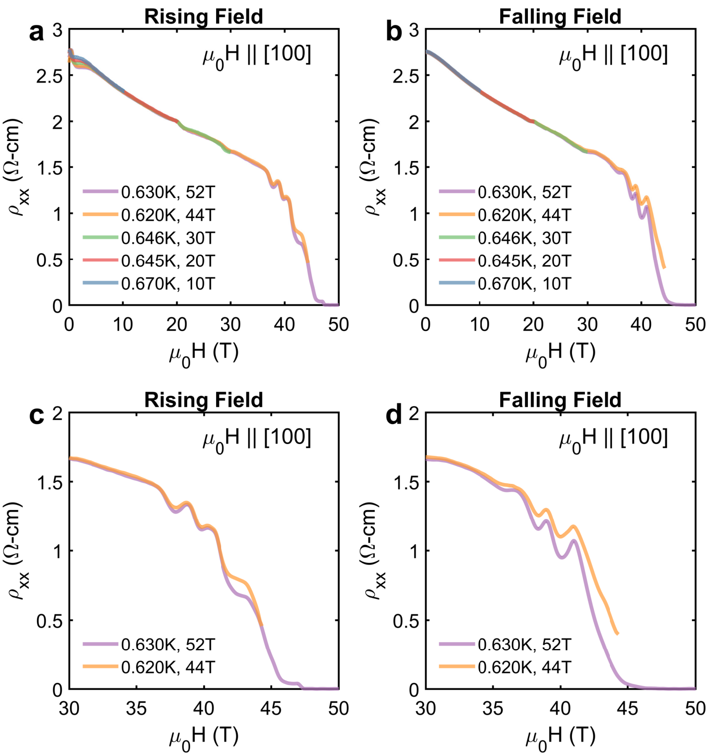

Supplementary Figure 16 Field-induced heating effects were assessed by using pulses with different maximum fields at the same temperature. The (a) rising and (b) falling field data for these pulses indicate minimal field-induced heating below $\sim$ 35T. There are small differences in (c) the rising field data above $\sim$ 42T and (d) the falling field data above $\sim$ 35T. These effects have a minimal impact on the positions of the quantum oscillations.

below $\sim$ 35T is independent of the maximum field. The rising field data possesses slight differences above $\sim$ 42T, as does the falling field data above $\sim$ 35T. However, directly comparing the rising and falling field data for the 44T and 52T pulses (Supplementary Fig. 17) shows the position of the quantum oscillations is minimally impacted by these effects. To further minimize the impact of these effects on our conclusions, where possible, we limited ourselves to analyzing data acquired while the sample was immersed in liquid and from pulses with similar maximum fields.

It is worth noting that there is a small, and systematic, difference between the positions of the quantum oscillations in the rising and falling field data even when the maximum field is chosen to keep the sample within the insulating state (*e.g.*, Supplementary Fig. 17). This shift is small and does not affect our conclusions, but its origin is unclear. Eddy current heating of the electrodes (unlikely) or magnetocaloric effects [23] in the sample close to the transition are possible explanations. Note, these effects are present even when the maximum

field is kept below the insulator-metal transition, precluding effects stemming from entry into the metallic state (*e.g.*, hysteretic effects).

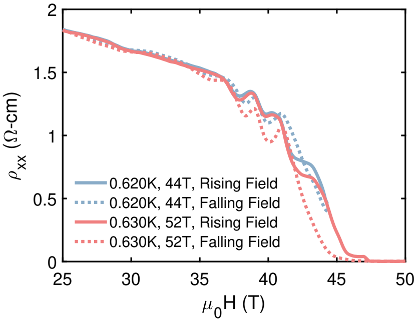

Supplementary Figure 17 There is a small, systematic shift between the positions of the quantum oscillations during the rising and falling fields. This shift occurs even when the sample does not enter the field-induced metallic state.

Supplementary Note 14: Checks for Joule Heating Effects

Joule heating effects were analyzed by varying the current used for resistivity measurements during a sequence of 60T pulses at similar temperatures and a fixed angle. As shown in Supplementary Fig. 18, Joule heating in the insulating state causes the measured resistivity to shift to lower values for fixed magnetic fields. This trend is consistent with the temperature dependent magnetoresistance shown in Fig. 1 of the main text. These effects do not alter the position or shape of the quantum oscillations in an appreciable manner, however we utilized the smallest current possible to minimize impacts of Joule heating.

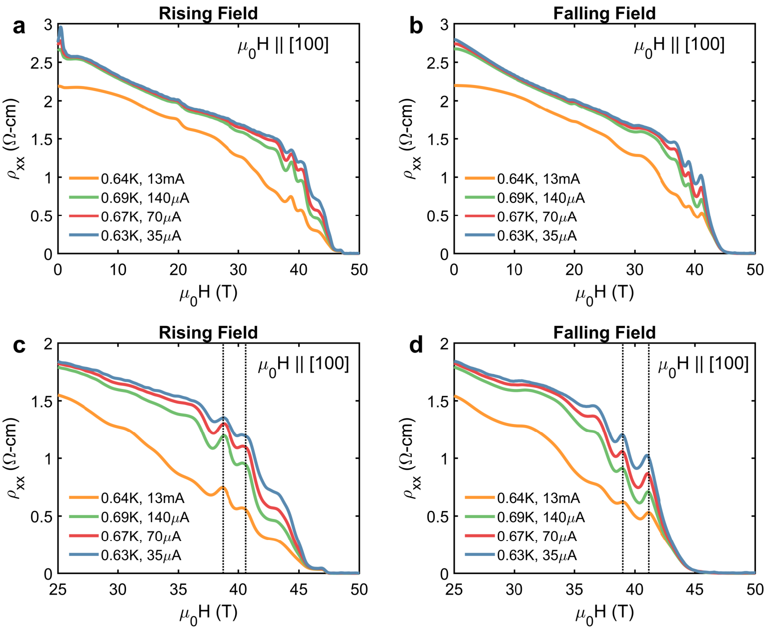

Supplementary Figure 18 Joule heating effects were assessed by varying the current used to measure the resistivity during the pulsed field experiments. For a given field, an increase in current caused a reduction in resistivity in both the (a) rising field and (b) falling field, consistent with Joule heating. Importantly, the positions and shapes of the quantum oscillation in both the (c) rising field and (d) falling field are minimally affected. Vertical dashed lines are guides for the eye.

References

[1] Iga, F., Shimizu, N. & Takabatake, T. Single crystal growth and physical properties of kondo insulator YbB_{12}. *J. Magn. Magn. Mater.* **177-181**, 337–338 (1998). https://doi.org/10.1016/S0304-8853(97)00493-9 .

[2] Riseborough, P. S. Heavy fermion semiconductors. *Adv. Phys.* **49** (3), 257–320 (2000). https://doi.org/10.1080/000187300243345 .

[3] Xiang, Z. *et al.* Quantum oscillations of electrical resistivity in an insulator. *Science* **362** (6410), 65–69 (2018). https://doi.org/10.1126/science.aap9607 .

[4] Sato, Y. *et al.* Topological surface conduction in Kondo insulator YbB_{12}. *J. Phys. D: Appl. Phys.* **54** (40), 404002 (2021). https://doi.org/10.1088/1361-6463/ac10d9 .

[5] Aoki, D., Knafo, W. & Sheikin, I. Heavy fermions in a high magnetic field. *Comptes Rendus Physique* **14** (1), 53–77 (2013). https://doi.org/10.1016/j.crhy.2012.11.004 .

[6] Mott, N. Rare-earth compounds with mixed valencies. *Philosophical Magazine* **30**, 403–416 (1974). URL https://www.tandfonline.com/doi/abs/10.1080/14786439808206566. https://doi.org/10.1080/14786439808206566 .

[7] Liu, H. *et al.* Fermi surfaces in Kondo insulators. *J. Phys. Condens. Matter* **30** (16), 16LT01 (2018). https://doi.org/10.1088/1361-648X/aaa522 .

[8] Knolle, J. & Cooper, N. R. Quantum Oscillations without a Fermi Surface and the Anomalous de Haas–van Alphen Effect. *Phys. Rev. Lett.* **115**, 146401 (2015). https://doi.org/10.1103/PhysRevLett.115.146401 .

[9] Knolle, J. & Cooper, N. R. Excitons in topological kondo insulators: Theory of thermodynamic and transport anomalies in SmB_6. *Phys. Rev. Lett.* **118**, 096604 (2017). https://doi.org/10.1103/PhysRevLett.118.096604 .

[10] Xiang, Z. *et al.* Hall Anomaly, Quantum Oscillations and Possible Lifshitz Transitions in Kondo Insulator YbB_{12}: Evidence for Unconventional Charge Transport. *Phys. Rev. X* **12**, 021050 (2022). https://doi.org/10.1103/PhysRevX.12.021050 .

[11] Xiang, Z. *et al.* Unusual high-field metal in a Kondo insulator. *Nat. Phys.* **17** (7), 788–793 (2021). https://doi.org/10.1038/s41567-021-01216-0 .

[12] Shoenberg, D. *Magnetic Oscillations in Metals* Cambridge Monographs on Physics (Cambridge University Press, 1984).

[13] Sugiyama, K., Iga, F., Kasaya, M., Kasuya, T. & Date, M. Field-Induced Metallic State in YbB_{12} under High Magnetic Field. *J. Phys. Soc. Japan* **57** (11), 3946–3953 (1988). https://doi.org/10.1143/JPSJ.57.3946 .

[14] Terashima, T. T. *et al.* Magnetization Process of the Kondo Insulator YbB_{12} in Ultrahigh Magnetic Fields. *J. Phys. Soc. Japan* **86** (5), 054710 (2017). https://doi.org/10.7566/JPSJ.86.054710 .

[15] McCollam, A., Fu, M. & Julian, S. R. Lifshitz transition underlying the metamagnetic transition of UPt_3. *J. Phys. Condens. Matter* **33** (7), 075804 (2020). https://doi.org/10.1088/1361-648X/abc729 .

[16] van Ruitenbeek, J. M. *et al.* A de Haas-van Alphen study of the field dependence of the Fermi surface in $ZrZn_2$. *J. Phys. F: Metal Physics* **12** (12), 2919 (1982). https://doi.org/10.1088/0305-4608/12/12/022 .

[17] McCollam, A., Andraka, B. & Julian, S. R. Fermi volume as a probe of hidden order. *Phys. Rev. B* **88**, 075102 (2013). https://doi.org/10.1103/PhysRevB.88.075102 .

[18] Ashcroft, N. & Mermin, N. *Solid State Physics* (Cengage Learning, 2011).

[19] Kushwaha, S. K. *et al.* Magnetic field-tuned Fermi liquid in a Kondo insulator. *Nature Communications* **10** (1), 5487 (2019). https://doi.org/10.1038/s41467-019-13421-w .

[20] Liu, H. *et al.* f-electron hybridised Fermi surface in magnetic field-induced metallic YbB_{12}. *npj Quantum Mater.* **7** (1), 12 (2022). https://doi.org/10.1038/s41535-021-00413-7 .

[21] Pippard, A. *The Dynamics of Conduction Electrons* (Gordon and Breach., 1965).

[22] Harrison, N. Highly Asymmetric Nodal Semimetal in Bulk SmB_6. *Phys. Rev. Lett.* **121**, 026602 (2018). https://doi.org/10.1103/PhysRevLett.121.026602 .

[23] Terashima, T. T. *et al.* Magnetic-Field-Induced Kondo Metal Realized in YbB_{12}. *Phys. Rev. Lett.* **120**, 257206 (2018). https://doi.org/10.1103/PhysRevLett.120.257206 .
